# Segway 2.0: Gaussian mixture models and minibatch training

**DOI:** 10.1093/bioinformatics/btx603

**Published:** 2017-09-22

**Authors:** Rachel C W Chan, Maxwell W Libbrecht, Eric G Roberts, Jeffrey A Bilmes, William Stafford Noble, Michael M Hoffman

**Affiliations:** 1 Princess Margaret Cancer Centre, Toronto, ON, Canada; 2 Engineering Physics Program, University of British Columbia, Vancouver, BC, Canada; 3 Department of Computer Science and Engineering, University of Washington, Seattle, WA, USA; 4 Department of Electrical Engineering, University of Washington, Seattle, WA, USA; 5 Department of Genome Sciences, University of Washington, Seattle, WA, USA; 6 Department of Computer Science, University of Toronto, Toronto, ON, Canada; 7 Department of Medical Biophysics, University of Toronto, Toronto, ON, Canada

## Abstract

**Summary:**

Segway performs semi-automated genome annotation, discovering joint patterns across multiple genomic signal datasets. We discuss a major new version of Segway and highlight its ability to model data with substantially greater accuracy. Major enhancements in Segway 2.0 include the ability to model data with a mixture of Gaussians, enabling capture of arbitrarily complex signal distributions, and minibatch training, leading to better learned parameters.

**Availability and implementation:**

Segway and its source code are freely available for download at http://segway.hoffmanlab.org. We have made available scripts (https://doi.org/10.5281/zenodo.802939) and datasets (https://doi.org/10.5281/zenodo.802906) for this paper’s analysis.

**Supplementary information:**

Supplementary data are available at *Bioinformatics* online.

## 1 Introduction

Segway identifies recurring combinatorial patterns in multiple genome-wide signal datasets such as ChIP-seq or DNase-seq data (Hoffman *et al.*, 2012). Segway uses discovered patterns to assign a label to every position in the genome, resulting in a semi-automated genome annotation. It is commonly used to define chromatin state across the whole genome by resources such as the ENCODE Project (ENCODE Project Consortium, 2012) or the Ensembl Regulatory Build (Zerbino *et al.*, 2015). Using chromatin data, the labels might represent genomic features such as ‘enhancer’ or ‘facultative heterochromatin’.

Since its initial publication, we have made many changes to Segway. Release notes (https://bitbucket.org/hoffmanlab/segway/src/default/NEWS) contain a complete list. Of the new features, we expect the new standalone mode to interest the most users. This mode removes the requirement for a cluster system such as Sun Grid Engine, allowing one to run Segway easily on any Linux host. Also of interest are new features which improve Segway’s ability to learn more complex patterns with less configuration. Below we describe these features and demonstrate the improvement they provide.

## 2 Results

### 2.1 Minibatch training

Segway uses the expectation-maximization (EM) algorithm to train its statistical model. Segway previously allowed for training only on a fixed region of the genome, such that each iteration of EM training uses the same fixed region. Using minibatch learning, each EM training iteration can now train on a different random region of the genome. For example, using a minibatch fraction of 1%, each training iteration will now use a different random 1% of the genome. This eliminates concerns of overfitting to a fixed region, but since there is no longer any guarantee of convergence, the final set of emission parameters is chosen by evaluating the likelihood on a held-out validation set. In general, minibatch allows one to sample the whole genome without having to use the whole genome as the training set, which would be ∼100 times slower. Using a smaller training region, however, does not affect RAM requirements. Segway performs inference on chunks of a fixed size. Therefore, using a larger training region amounts to simply using more chunks. Since the minibatch feature selects a different random region of the genome to train on per iteration, there is also a very high variation in likelihood progression between instances, though the overall likelihood progression is, however, generally more positive than that for a fixed region.

**Fig. 1:**
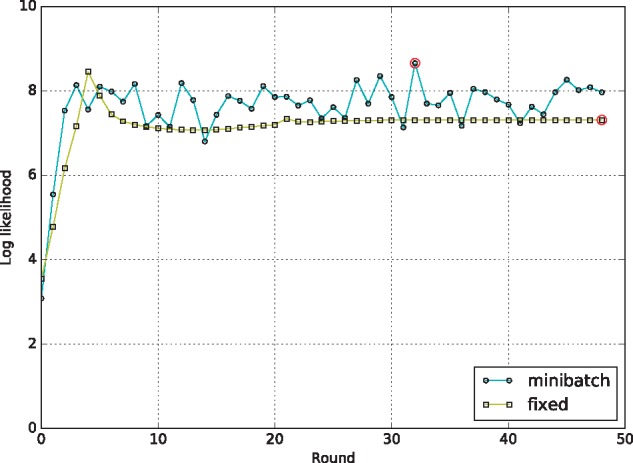
Log likelihood progression against round for a fixed 1% of the genome and a 1% chosen fraction for minibatch. Each series shared the same set of starting parameters and were evaluated against the same held-out validation set. Red circles: likelihood for the final chosen set of parameters in each series (Color version of this figure is available at *Bioinformatics* online.)

To demonstrate, we trained Segway on ENCODE Project Consortium (2012) GRCh38/hg38 ChIP-seq datasets for H3K4me1, H3K4me3, H3K27ac, H3K27me3 and CTCF in the DOHH2 cell line (Kluin-Nelemans *et al.*, 1991) (Supplementary Table S1). We did this using a single Gaussian model for a randomly selected fixed region of size 1%, and using minibatch training with batch size 1%. For each training round, we evaluated the posterior log likelihood of its learned parameters on 1.5% of the genome, which we held out from training in all cases. Minibatch resulted in a higher log likelihood convergence on the validation set both on average and in the final winning set of parameters (Fig. 1). The fixed case also suffered from the validation set likelihood dropping from its initial peak due to overfitting on the training set (Fig. 1).

### 2.2 Gaussian mixture models

Segway learns Gaussian distributions over signal values to represent different patterns. Previously, Segway used a single-component Gaussian to model the signal in each dataset given some label such that there is one learned mean parameter for each track-label pair, and one fixed variance for a given track. To enable more complex signal distributions, we extended Segway’s model to allow for a mixture model with *k* Gaussian components. Now, there are *k* mean parameters for each track-label pair, and *k* variances for each track. Using a mixture of Gaussians allows learning emission distributions that can more accurately fit data distributed non-normally.

**Fig. 2:**
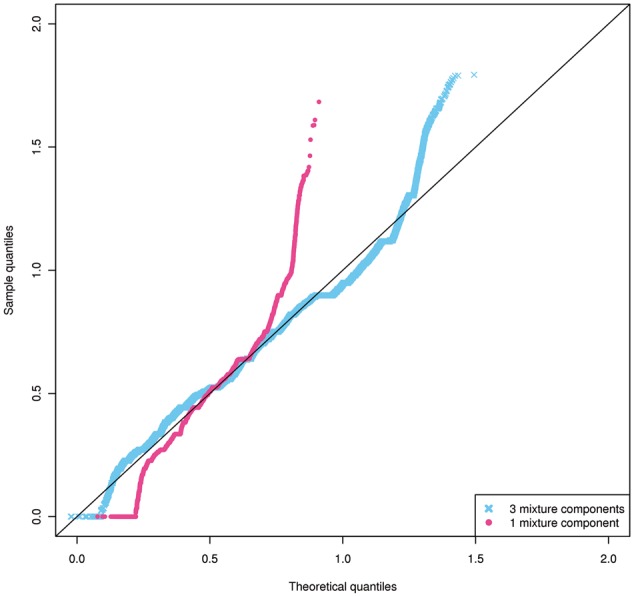
Combined quantile-quantile plot demonstrating ability of 1 or 3 Gaussian components to capture their empirical distributions

To demonstrate, we trained Segway on signal data for the histone mark H3K27ac in the cell line DOHH2, using a one-component Gaussian model and using a three-component mixture of Gaussians. As previously described in Roberts *et al.* (2016), we trained using minibatch on 1% of the genome, for 10 labels and 100 EM training iterations. For each label learned, we extracted all datapoints corresponding to that label in the final annotation to generate an empirical distribution. We also extracted a theoretical distribution from the model. We measured the match between a label’s theoretical and empirical distributions using the Kolmogorov-Smirnov statistic *D*. The smaller the *D* statistic, the closer the fit between the two distributions. For both the one- and three-component models, we identified the label with the lowest *D* and compared its theoretical distribution to its empirical distribution (Fig. 2). Because Segway performs unsupervised learning, the sets of labels between each case do not correspond identically.

In the single-component Gaussian case, the average *D* statistic across all labels was 0.28, with a median of 0.29, and a best *D* statistic of 0.078. In the three-component mixture of Gaussians case, the average *D* statistic across all labels was 0.16, with a median of 0.10, and a best *D* statistic of 0.058.

The theoretical distribution for the three-component mixture of Gaussians agreed with its multi-modal empirical distribution except for a slight right-skew in the data (Fig. 2 and Supplementary Fig. S1). In comparison, the theoretical distribution for the single-component Gaussian model does not agree very well with its empirical distribution, with a strong skew in the tails of the distribution (Fig. 2).

In conclusion, the mixture of Gaussians model better captures the empirical distribution than the single-component Gaussian model both on average and overall.

To examine how Gaussian mixtures affect how discovered patterns match biological features, we trained Segway 2.0 using one to five mixture components on ENCODE GRCh37/hg19 data. Specifically, we used DNase-seq data and ChIP-seq datasets for H3K27ac, H3K27me3, H3K36me3 and H3K4me3 in the cell line K562 (Supplementary Table S1). We used minibatch training (1% of genome) with a held-out validation set totaling 1% of the genome.

After producing a genome-wide Segway annotation, we used it to discriminate between active and inactive transcription start sites (TSSs) in K562, as in Hoffman *et al.* (2012). We identified the segments that overlapped the most upstream TSS of each GENCODE (Harrow *et al.*, 2012) gene. We defined positive predictions as those that overlapped a TSS with cytosolic poly(A)^+^ CAGE support in K562, and negative predictions as those that overlapped a TSS with no CAGE support. We evaluated the precision and recall obtained for all labels across 10 random starts for each number of mixture components (Supplementary Fig. S2). For each random start and number of mixture components, we identified the label with the best precision, and we calculated that label’s recall. The one-component mixture achieved a mean ± SD best precision of 0.49 ± 0.05, and a corresponding recall of 0.49 ± 0.21. The five-component mixture achieved a best precision of 0.42 ± 0.03, and a corresponding recall of 0.74 ± 0.15. This represents a small but significant difference in precision (Wilcoxon rank-sum test; *P* = 0.0009) but a large significant increase in recall (Wilcoxon rank-sum test; *P* = 0.003).

### 2.3 Comparison with other methods

Segway 2.0’s design features distinguish it from methods such as ChromHMM (Ernst *et al.*, 2012) and Segway 1.0 (Table 1). To compare computational performance, we benchmarked Segway 2.0.1, Segway 1.3.0 and ChromHMM 1.12 on a dedicated host with two eight-core Intel Xeon E5-2650v2 CPUs (2.60 GHz) with 229 GiB of memory and hyperthreading, virtualized by QEMU as 32 virtual CPUs (Table 2). We limited each program to eight processes. We performed the five-dataset training procedure outlined earlier, without validation, and limited to 10 training rounds. ChromHMM does not support the bigWig format we used for Segway. Instead, we merged the Binary Alignment/Map (BAM) replicates originally used to generate these bigWig files. We then used ChromHMM’s BinarizeBam to create intermediate files before starting the benchmark. For each program, we set the same resolution (10 bp) and number of training rounds (10) to avoid performance differences solely due to these parameters.

**Table 1.** Major differences in design features between ChromHMM, Segway 1.0 and Segway 2.0, adapted from Hoffman *et al.* (2013)

	ChromHMM	Segway 1.0	Segway 2.0
Modeling framework	Hidden Markov model	Dynamic Bayesian network	Dynamic Bayesian network
Default genomic resolution	200 bp	1 bp	1 bp
Handling missing data	Boolean	Real value	Real value
Emission modeling	Bernoulli distribution	Gaussian distribution	Gaussian **mixture model**
Length modeling	Geometric distribution	Geometric plus hard and soft constraints	Geometric plus hard and soft constraints
Training set	Entire genome	Fixed regions	**Minibatch or** fixed regions
Decoding algorithm	Posterior decoding	Viterbi	Viterbi

*Note*: Bold text: additions in Segway 2.0.

**Table 2.** Time, memory and disk space used by ChromHMM, Segway 1.3 and Segway 2.0 to train 10 rounds on a single histone modification ChIP-seq dataset at 10 bp resolution, with the default training region for each method

	ChromHMM 1.12	Segway 1.3.0	Segway 2.0.1
Genomic resolution	10 bp (default 200 bp)	10 bp (default 1 bp)	10 bp (default 1 bp)
Training rounds	10 (default 200)	10 (default 100)	10 (default 100)
Training region	100% of genome	1% of genome (fixed)	1% of genome (minibatch)
Wall time (hh: mm: ss)	00: 20: 23 ± 00: 00: 20	00: 24: 38 ± 00: 00: 02	00: 22: 15 ± 00: 00: 12
Total CPU time (hh: mm: ss)	01: 22: 26 ± 00: 07: 03	01: 11: 51 ± 00: 00: 49	01: 41: 40 ± 00: 01: 07
Max resident set size (GiB)	31.0 ± 1.2	1.6 ± 0.0001	4.7 ± 0.002
Max virtual memory (GiB)	59.2 ± 0.07	2.3 ± 0.000002	9.0 ± 0
Input disk space (GiB)	2.9	3.0	3.0
Working disk space (MiB)	0.14 ± 0	123.0 ± 0.00007	1.2 ± 0.005

*Note*: Reported values are mean ± SD for three trials. SD of 0 is exact.

ChromHMM and Segway 2.0 completed training in similar wall (20–25 min) and CPU times (1–2 h). To train at 10 bp resolution, ChromHMM required a large amount of RAM (31.0 GiB), making it impossible on most current workstations. Segway 2.0 only required 4.7 GiB of RAM. Segway 2.0’s efficient observation storage reduces considerably working disk space to only 1.2 MiB, compared with Segway 1.3 (123.0 MiB). The new storage system increases CPU time but decreases overall wall time from Segway 1.3. It also enables minibatch training across the whole genome and the better models that result.

## Supplementary Material

Supplementary Data
