# Supplementary material for: Segway 2.0: Gaussian mixture models and minibatch training
Source: Bioinformatics. 2017 Sep 22;34(4):669–71. doi: 10.1093/bioinformatics/btx603 (PMC5860603; doi:10.1093/bioinformatics/btx603)
Supplement: Supplementary Data [file btx603_supplement.pdf]

Supplemental figures

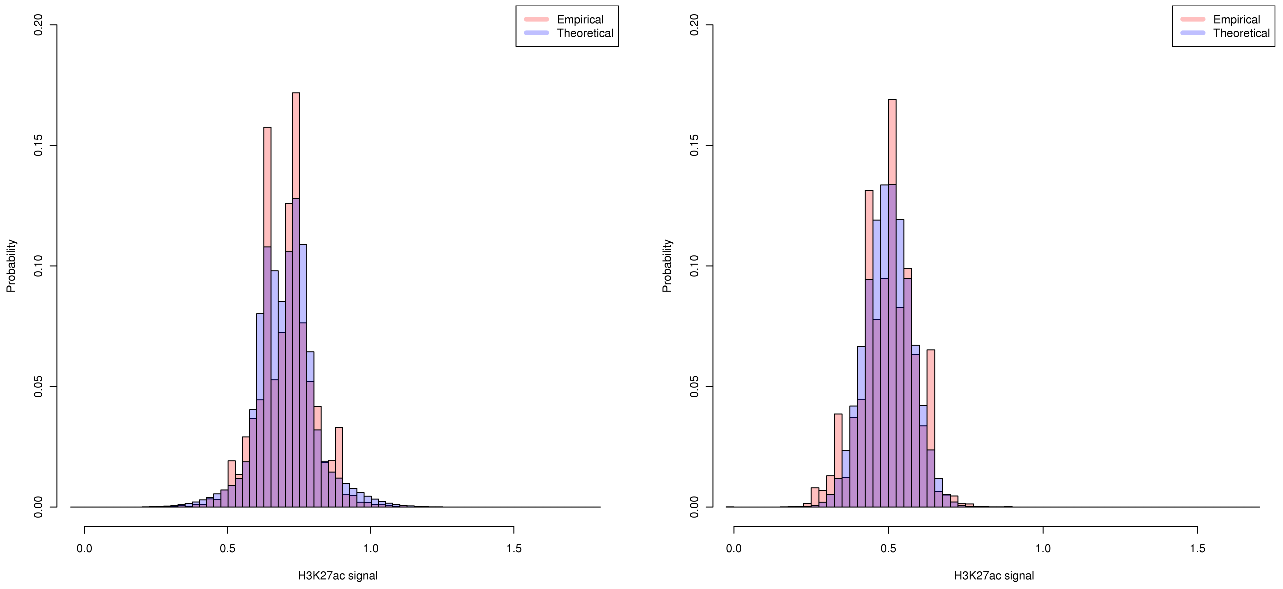

(a) 3-component (b) 1-component

Fig. S1. Histograms for the 3 (left) and 1 (right)-component mixtures of Gaussians showing the labels with the best D statistic in each case. The histograms are between the datapoints underneath that label (pink bins) against the same number of datapoints drawn randomly from the theoretical distribution of that label (blue bins).

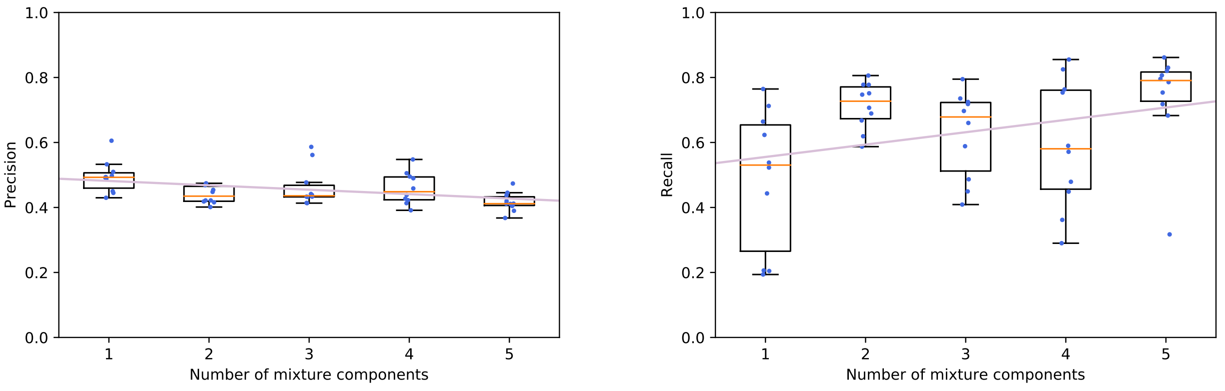

(a) Precision of the label with best precision (b) Recall of the label with best precision

Fig. S2. Boxplots of (a) precision and (b) recall for the label with top precision for active transcription start sites (TSSs) in K562 ($N = 50$). For each of models with 1–5 mixture components, and each of 10 random starts for each number of components, we plotted precision and recall for the label with the highest precision. Orange line: median. Black box: interquartile range (IQR). Black whisker: most extreme value within quartile $\pm 1.5\,$IQR. Blue point: individual label. Grey line: regression line.

Supplemental table

Assembly	Cell type	Assay	ENCODE accession IDs[a] or filename[b]
**Minibatch training & Comparison with other methods — Segway**			
GRCh38/hg38	DOHH2	ChIP-seq: H3K4me1	ENCFF509XSM
		ChIP-seq: H3K4me3	ENCFF745GML
		ChIP-seq: H3K27ac	ENCFF890NAY
		ChIP-seq: H3K27me3	ENCFF592CSV
		ChIP-seq: CTCF	ENCFF884IIL
**Comparison with other methods — ChromHMM**			
GRCh38/hg38	DOHH2	ChIP-seq: H3K4me1	ENCFF453IVZ, ENCFF768TYB
		ChIP-seq: H3K4me3	ENCFF131YRA, ENCFF585ZNY
		ChIP-seq: H3K27ac	ENCFF079AQF, ENCFF479VLR
		ChIP-seq: H3K27me3	ENCFF133EQW, ENCFF370MOM
		ChIP-seq: CTCF	ENCFF092CZO, ENCFF863PSQ
**Gaussian mixture models (distribution comparison)**			
GRCh38/hg38	DOHH2	ChIP-seq: H3K27ac	ENCFF890NAY
**Gaussian mixture models (active TSS prediction)**			
GRCh37/hg19	K562	ChIP-seq: H3K4me3	wgEncodeBroadHistoneK562H3k4me3StdAln_2Reps.norm5.rawsignal.bw
		ChIP-seq: H3K27ac	wgEncodeBroadHistoneK562H3k27acStdAln_2Reps.norm5.rawsignal.bw
		ChIP-seq: H3K27me3	wgEncodeBroadHistoneK562H3k27me3StdAln_2Reps.norm5.rawsignal.bw
		ChIP-seq: H3K36me3	wgEncodeBroadHistoneK562H3k36me3StdAln_2Reps.norm5.rawsignal.bw
		DNase-seq	wgEncodeUwDnaseK562Aln_2Reps.norm5.rawsignal.bw
		CAGE: cytosolic long poly(A)$^+$ RNA	ENCFF983UJK, ENCFF095ZKV

Table S1. Datasets used.

[a] ENCODE accession IDs are available at https://www.encodeproject.org/experiments/.

[b] Filenames are available in the directory ftp://ftp.ebi.ac.uk/pub/databases/ensembl/encode/integration_data_jan2011/byDataType/signal/jan2011/bigwig/.
